# Testing and Evaluation of Flexural Tensile Strength of Prestressed CFRP Cables

**DOI:** 10.3390/ma15207065

**Published:** 2022-10-11

**Authors:** Jiajun Xia, Zhirong Xu, Ping Zhuge, Bing Wang, Wanyun Cai, Jiaping Fu

**Affiliations:** 1 School of Civil and Environmental Engineering, Ningbo University, Ningbo 315211, China; 2 Ningbo Communications Planning Institute Co., Ltd., Ningbo 315000, China

**Keywords:** bridge engineering, CFRP cables, bending tensile strength, prestressing force, steering systems

## Abstract

To expand the application scope of prestressed carbon fiber-reinforced polymer (CFRP) cables in civil engineering, the ultimate tensile strength of these cables was tested and evaluated under bending conditions. First, the study analyzed the tensile failure mechanism of CFRP cables under bending conditions based on elastic bending analysis theory. Thereafter, the ultimate stress state of individual tendons and cables was derived and a calculation model for the tensile strength of bent CFRP cables was established. Second, 14 sets of test conditions were created for CFRP cables under bending angles of 20–40° and bending radii of 1.5–3 m. Then, bending tensile tests were conducted to evaluate the effects of the above factors on the ultimate tensile strength, and the correctness of the computational model was verified using experiments. Finally, the ultimate performance of CFRP cables was theoretically predicted using the established model. The results showed that the cable bending tensile strength was associated with the radius *r*, tensile strength *f*, and elastic modulus *E* of the reinforced material and the bending radius *R,* but was not correlated with the interface buffer material or the bending angle of the steering system. Moreover, the flexural tensile residual strength was only affected by *R/r* and *E/f*. When *E/f* involved conventional material parameters, the residual strength increased nonlinearly with increased *R/r*. When *R/r* ≥ 600, the residual strength reached more than 80%. Therefore, *R/r* at 600 could be used as the design basis for a safe critical radius.

## 1. Introduction

Carbon fiber-reinforced polymer (CFRP) is recognized in engineering as an ideal substitute for traditional steel tendons in harsh environments [1,2,3,4,5] due to its high strength, light weight, and high corrosion resistance, which can effectively reduce structural weight and improve structural durability [6,7,8]. In the 1980s, Japan, the United States, and other countries began to study its engineering applications [9,10] and the U.S. Specification ACI 440.4R-04 [11] recommends the use of CFRP cables as a prestressing material. However, in potential practical engineering applications, such as the flexural reinforcement of simply supported beams with external prestressed CFRP cables [12,13,14] and CFRP cables spanning cable saddles of suspension bridge [15,16,17], CFRP cables need to be bent at a certain angle [18,19,20]. Traditional materials tend to use steel strands and cables as members, which have less performance compromise from bending due to the superior ductility and plastic deformation of steel. This is one of the reasons as to why such problems are rarely studied in depth. Different from steel, because CFRP materials are brittle, the unidirectional arrangement of carbon fiber precursors in the matrix leads to poor lateral mechanical properties in such materials [21,22,23]. Further studies describe a significant decline in the mechanical properties of CFRP cables after bending [24,25], which is also one of the leading constraints on its development. Therefore, it is particularly important to evaluate the ultimate tensile strength of CFRP cables in a bent state.

At present, research on the tensile properties of CFRP cables in a linear state is relatively mature, especially regarding the material mechanical properties [11,26] and anchoring system [27,28]. In this regard, they have demonstrated good results in practical applications [29].

With the continued development of CFRP cables, more and more scholars have begun to pay attention to their tensile properties in a bent state and have carried out related studies. Jerrett et al. [24] conducted the first performance test of CFRP tendons under bending behavior (with a turning radius of 406 mm) in 1996 and proposed the related basic hypothesis. It was found that the outer fiber strain of CFRP tendons far exceeds the central axial strain, causing cumulative damage that may further reduce the strength. Adachi et al. [30] and Jerrett et al. [31] conducted an experimental study by increasing the turning radius to almost 510 mm and found that the turning radius has a large effect on the strength of CFRP tendons. The American Concrete Institute (ACI) [32] and the Society for Testing and Materials (ASTM) [33,34] proposed a test method for assessing the tensile strength of CFRP tendons under bending, applicable to a situation in which tendons are bent in use. El-Sayed et al. [35] found in their experimental research that the tensile strength of CFRP tendons in a bending state is substantially reduced when the bending radius is small. Menezes et al. [36] developed a finite element model to simulate the mechanical properties of CFRP tendons under tensile and four-point bending loads. Ping Z et al. [37] tested the ultimate bending load of CFRP tendons under three bending radii and presented curves of the effects of the bending radius and diameter of tendons on tensile strength. Zhang et al. [38] designed a four-point loading bending test for CFRP tendons with various diameters, studied the relationship between the outer fiber strain and load, and recommended the design value for the bending tensile strength based on the test results. Through experimental research, Sami et al. [39] put forward an empirical formula for the bending tensile strength of CFRP tendons with a small bending radius.

In summary, the research results on the bending tensile strength of CFRP tendons are mainly derived from experiments or finite element simulations, but the theories on the evaluation of their failure behavior under bending are not perfect, and the failure mechanism is not yet clear. This is especially true for theoretical and experimental studies on the evaluation of the performance of cords under bending, as to which there are few published reports and further research is needed.

In order to further reduce the impact of bending on the performance of CFRP ropes and to expand the application scope of prestressed CFRP cables in civil engineering, this paper conducts an exploratory study for the working conditions with a turning radius greater than 2000 mm, which has not been studied by scholars before. Moreover, an analytical and evaluation model for the tensile strength of CFRP cables under bending conditions was established through theoretical analysis and experimental verification, and the cable residual strength under different bending conditions was predicted using the model. First, the internal force failure criterion was employed to analyze the bending and tensile failure behavior of individual CFRP tendons and cables, to determine the failure mechanism, and to establish an evaluation calculation model. Second, based on different bending radii, bending angles, and interfacial buffer materials, 14 sets of working conditions were established to conduct experimental research and verify the correctness of the calculation model. Finally, the ultimate bearing capacity (UBC) and flexural tensile residual strength were predicted theoretically using the model. These research achievements can be used in the evaluation and reference of prestressed CFRP cable engineering applications.

## 2. Theoretical Research

### 2.1. Failure Mechanism

**Figure 1:**
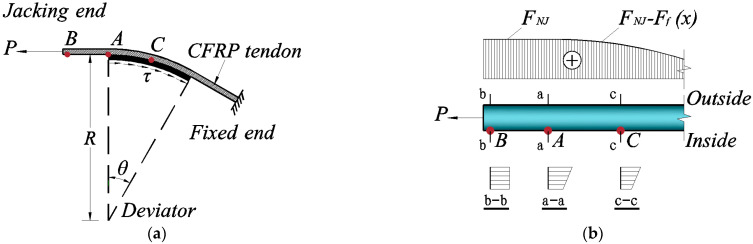
(**a**) Overall force of a single tendon, (**b**) Force of the cross-section of a single tendon. Stress analysis of CFRP tendons under bending.

CFRP tendons generally use a steering system to achieve overall bending steering and the CFRP tendons in a bending state are taken for the force analysis (Figure 1a). The following basic assumptions were made [24]: (1) Under the limit state, the cross-section satisfies the assumption of a plane cross-section; (2) cross-sectional changes due to axial stress are ignored; and (3) the CFRP fiber strands are always in an elastic state in the axial direction.

A schematic diagram of the overall force of a CFRP tendon during turning is shown in Figure 1a, where *θ* represents the tendon bending angle, *R* the bending radius of the turning block, A the tangent point at which the tendon enters into the bogie, B the point of the tension end, and C any point on the turning segment. When the left end of the tendon was placed under tension by load *P*, the tendon on the steering block was subjected to a rightward frictional force *F_f_(x)*. At this time, the local force of the tendons at the bogie involved the axial force of tendons, which remained unchanged from point B at the tension end to point A entering the bogie (Figure 1b). The axial force continuously decreased from points A to C due to the existence of the friction force on the bogie interface (*F_NJ_* − *F_f_(x)*). At the same time, the elongation values of the inner and outer fibers of the tendons on the bogie were different and there was a strain difference, which led to different stress distributions in the cross-section at point B (section b-b), point A (section a-a), and point C (section c-c). The outer stress of the section at point A was then the maximum, such that the section at point A could be used as the CFRP reinforcement-control section under the limit state.

According to Ping Z et al. [37], the residual strength *f_tu_* after bending can be expressed as

(1) $$f_{tu}=f_{tk}-\frac{r}{R}E\;\;\;\left(R\geq \frac{2E}{f_{tu}}r\right)$$

For convenience, the ratio of the flexural tensile residual strength *f_tu_* to the material tensile strength *f_tk_* was defined as the flexural tensile strength retention rate *η_t_*, expressed as

(2) $$\eta_{t}=\frac{f_{tu}}{f_{tk}}\times 100\%=\left(1-\frac{E}{f_{tk}}\cdot \frac{r}{R}\right)\times 100\%$$

According to $E=\frac{f_{tk}}{\varepsilon_{u}}$, then $\varepsilon_{u}=\frac{f_{tk}}{E}$, where $\varepsilon_{u}$ represents the ultimate strain of the material. As mentioned above, there was a strain difference Δε caused by bending between the inner and outer sides and the strain increment $\varepsilon_{b}=\frac{1}{2}\Delta \varepsilon =\frac{1}{2}\cdot \frac{\theta (R+r)-\theta R}{\theta R}=\frac{r}{R}$ of the outer fibers compared to the fibers at the center of the circle obtained, such that Equation (2) was expressed as

(3) $$\eta_{t}=(1-\frac{\varepsilon_{b}}{\varepsilon_{u}})\times 100\%$$

The retention rate of flexural tensile strength *η_t_* is the major index for evaluating the flexural resistance of CFRP tendons, which can intuitively reflect the performance rate of the material. A larger *η_t_* indicates the higher performance of the material and better bending resistance.

### 2.2. Tensile Strength of CFRP Tendons

As mentioned above, Equation (2) is only applicable to the initial failure state of outermost fiber fracture and, in fact, for CFRP tendons with a general circular cross-section, the overall ultimate failure state has not yet been reached. With increased load, failure is layered. When outer fibers reach the ultimate tensile strength, the remaining fibers have not yet reached their ultimate tensile strength. After the initial failure of the tendon, the outer fibers fracture and are out of service, the stress redistributed, and the above process repeated until the overall limit state is reached.

**Figure 2:**
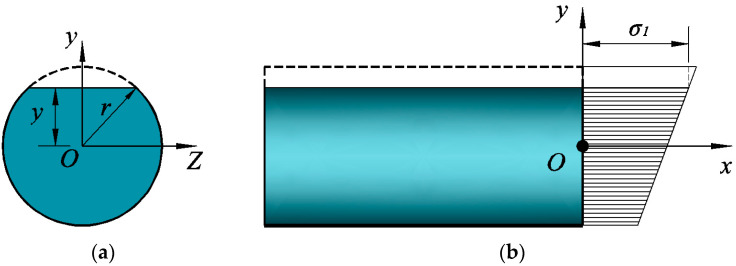
(**a**) Schematic diagram of control section, (**b**) Control section stress. Section stress analysis.

The cross-sectional state of CFRP tendons at the ultimate failure was assumed (Figure 2a) with the shaded area the effective working cross-section and the stress at the upper edge of the shaded area at *σ*_1_ at this time. The stress distribution of the section is shown in Figure 2b. The stress at the center of the circle is $\sigma_{1}-\frac{E}{R}y$, the stress of the full circular section is $(\sigma_{1}-\frac{E}{R}y)\cdot A$, and stress of the out-of-service section is approximately $\sigma_{1}A_{1}$, such that its effective bearing capacity in this state, *F*, could be expressed as

(4) $$F=(\sigma_{1}-\frac{E}{R}y)\cdot A-\sigma_{1}A_{1}$$

where, the circular full cross-sectional area *A* = π*r*^2^, the failure cross-sectional area $A_{1}=\arccos\left(\frac{y}{r}\right)\cdot r^{2}-\sqrt{r^{2}-y^{2}}\cdot y$

When the stress *σ*_1_ on the upper edge reached the ultimate strength of the material *f_tk_*, the tendons as a whole reached the ultimate failure state. In Equation (4), the effective bearing capacity *F* is a function of *y*. Based on the calculations, *F* was found to only have one maximum value within the range of y∈(0,r), which conformed to the actual failure relationship, such that the UBC was

(5) $$F_{\max}=(f_{tk}-\frac{E}{R}y)\cdot A-f_{tk}A_{1}$$

where, $y=r\cdot \sqrt{1-{\left(\frac{E\pi r}{2Rf_{tk}}\right)}^{2}}$.

Therefore, the residual strength retention (RSR) rate of bending was

(6) $$\eta =\frac{F_{\max}}{Af_{tk}}=(1-\frac{Ey}{Rf_{tk}}-\frac{A_{1}}{A})\times 100\%$$

In conclusion, after substitution and simplification, it was obtained that

(7) $$\eta =1-\frac{1}{2}\cdot \frac{\varepsilon_{b}}{\varepsilon_{u}}\sqrt{1-(\frac{\pi }{2}\cdot \frac{\varepsilon_{b}}{\varepsilon_{u}}{)}^{2}}-\frac{1}{\pi }\arccos\left(\sqrt{1-(\frac{\pi }{2}\cdot \frac{\varepsilon_{b}}{\varepsilon_{u}}{)}^{2}}\right)\;\left(\frac{\varepsilon_{b}}{\varepsilon_{u}}\leq \frac{2}{\pi }\approx 0.637\right)$$

where the material limit strain was $\varepsilon_{u}=\frac{f_{tk}}{E}$, the strain increment caused by bending $\varepsilon_{b}=\frac{r}{R}$.

At this time, the UBC under bending is $F_{tu}=\eta f_{tk}A$.

### 2.3. Tensile Strength of CFRP Cables

A CFRP cable is composed of multiple CFRP tendons and the bending radii of the tendons at different heights within the cable body are different under the bending state. In the same cross-section, there are slight differences in the strain distribution across tendons within the cable. Therefore, it is necessary to comprehensively analyze the stress–strain state of each single tendon to determine the ultimate failure mode, so as to be able to derive the theoretical evaluation model for the ultimate strength of CFRP cables in the bending tension state.

**Figure 3:**
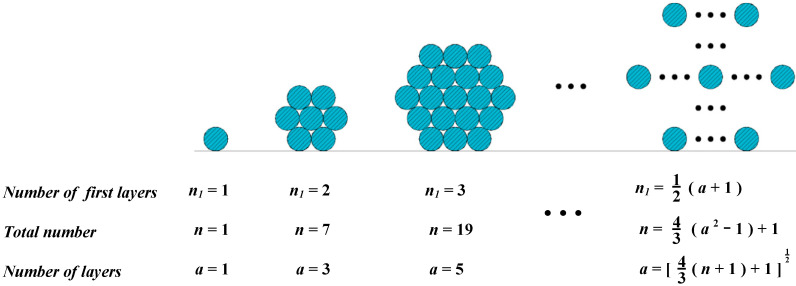
Arrangement of the CFRP cable section.

A section of a CFRP cable is a regular hexagon or close to a hexagon and is regularly arranged (Figure 3). The number of CFRP tendons in the first layer gradually increases from *n*_1_ = 1. According to the characteristics of the hexagonal section, the relationship between the number of the first layer *n*_1_, the total number *n*, and the number of layers *a* was calculated.

According to Equation (5), for each layer of tendons in the cable body, the value of *R* in the formula changes to *R +* (*i*−1) *d*. The UBC of each tendon within the cable body was assumed to be expressed by variables, including

(8) $$F_{\max}(i)=(f_{tk}-\frac{E}{R+(i-1)d}y)A-f_{tk}A_{1}$$

where *i* denotes the particular layer to which a single tendon belongs.

Then, the ultimate strength of each tendon within the cable body was expressed as

(9) $$f_{i}=\frac{F_{\max}(i)}{A}$$

According to Equations (11) and (12), the strengths of the tendons in the same layer are equal and the strength of the tendons of the first layer at the bottom is the lowest and then increased in turn outward.

Point A of the control section was assumed to be in ideal axial tension, with the tension force applied on each tendon within the parallel cable being the same, such that, when the load reached *nf*_1_, the tendons of the first layer with the lowest strength f1 failed and then became out of service, and the load redistributed to the remaining (*n* − *n*_1_) tendons. There were two failure modes:

Failure mode (1): If *nf_1_* > *(n − n_1_)f_2_*, the remaining *(n − n_1_)* tendons cannot bear the load and the tendons of the 2nd layer with the 2nd-lowest strength *f_2_* will fail and become out of service synchronously. Further, *nf_1_* and *(n−n_1_-n_2_)f_3_* are compared to judge the failure of tendons of the 3rd layer.

Failure Mode (2): If *nf1 < (n − n_1_)f_2_*, the remaining *(n − n_1_)* tendons can bear the load and more loads are applied. When the tendons of the 2nd layer with the 2nd-lowest strength *f_2_* failed and became out of service, *(n − n_1_)f_2_* and *(n − n_1_ − n_2_)f_3_* are compared to judge the failure of tendons of the 3rd layer.

The above steps are repeated until the judgment of failure of all tendons is completed and the equivalent ultimate strength of each tendon within the cable body obtained as

(10) $$f_{e}=\max\left\{nf_{1},\;(n-n_{1})f_{2},\;\ldots \;,\;f_{n}\right\}/n$$

Then, the UBC of the CFRP cables is *Fs = nAf.*

Considering the CFRP cables to be in the ideal axial tension situation, the critical number n corresponding to different *R*’s was obtained by calculation (Table 1). When *R* was 0.7 m, the ultimate failure mode of *n* < 5550 belonged to the first mode above, such that the first low-strength tendon failed and the remaining tendons could not bear the original load and continuously failed.

Considering the actual application scenario, the number *n* was much smaller than the data calculated in Table 1, such that Equation (10) was simplified to

(11) $$f_{e}=f_{1}$$

According to the failure criterion $P\geq F_{S}$ (maximum axial force failure criterion), the UBC of the CFRP cable was

(12) $$F_{s}=nAf_{1}$$

The strength retention rate under bending was the same as in Section 2.2 (Equation (7)), expressed as

(13) $$\eta =\frac{f_{1}}{f_{tk}}\times 100\%$$

## 3. Experimental Study

### 3.1. Tensile Strength Test of CFRP Cables

The reliability of the cable-anchor system used in this test was verified and the effects of anchorage performance ruled out using bending tests. A static load test of the CFRP cable was carried out to measure the true ultimate tensile strength of the test piece.

**Figure 4:**
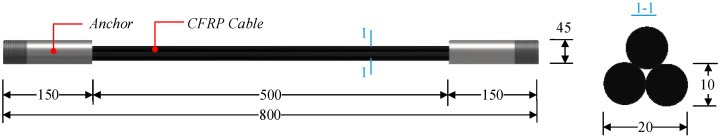
Test pieces of CFRP cable (in mm).

Three CFRP tendons of 10 mm diameter were used as test pieces. The length of the free end was 500 mm (Refer to American Standard ACI 440.3R-04: the length of the specimen should not be less than 40 times the diameter of the tendon), and the anchorage lengths at both ends were 150 mm. The specific sizes of the test pieces are shown in Figure 4.

During tests, the universal servo tensile testing machine was employed to apply load on the test pieces and the data related to the force, displacement, and failure mode of the cable were recorded. By reference to the Japanese standard JSCE-E 531-1995 [40], the following loading scheme was used. For anchorage assembly parts of the cable, preloading and unloading were performed with 5% of the standard value of the tensile strength of the CFRP material provided by the manufacturer. Then, step-by-step loading was performed with a load increment of 10% of the standard value of the tensile strength at a loading rate of ~150 MPa/min. Finally, loading was continued until complete failure to obtain the UBC.

**Figure 5:**
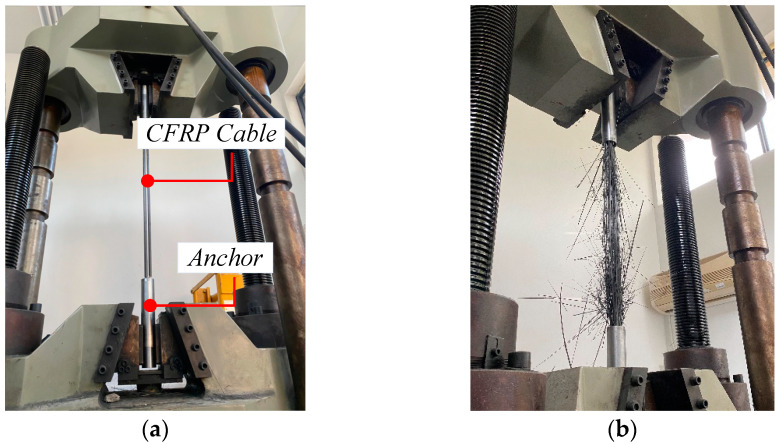
(**a**) Installation, (**b**) Failure. Static load test on the CFRP cable.

In this test, the only failure mode of the CFRP cable anchorage system was overall rupture failure of the cable body and “wire rupture” failure occurred in the free segment of the cable (Figure 5b). Based on the tensile tests of triplicate pieces, data on tensile UBC were obtained (Table 2).

The average test value of three test pieces was 492.1 kN. The ultimate strength of the CFRP cable *f_t_* was 2089 MPa by converting *F* = *A·f_t_*. The ultimate tensile strength provided by the CFRP material manufacturer was 2200 MPa. Therefore, the anchorage efficiency of the test pieces used in this test was 95.0%, which was consistent with that required by the standard.

### 3.2. Tensile Strength Test of CFRP Cable under Bending

#### 3.2.1. Test Scheme

A beam from a bridge that was over 25 years old was tested. The beam type was a hollow slab beam, 1000 × 600 × 6000 mm in size, with a concrete grade of C30 and without prestressed steel tendons. The overall condition was good.

**Figure 6:**
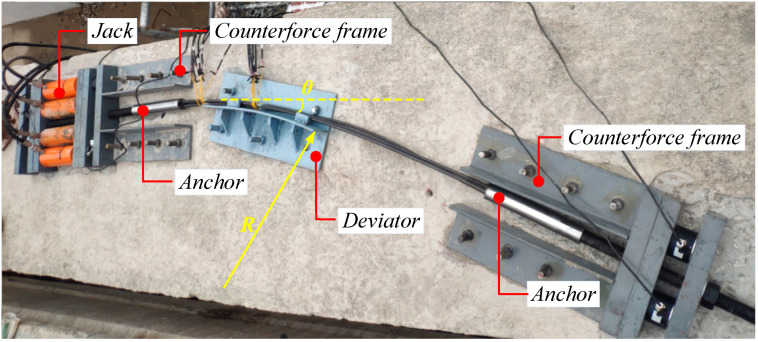
Tests of CFRP cables under bending.

The test used the external prestress tensioning technique, consisting of an anchorage, connection, steering system, tensioning system, and monitoring systems (Figure 6). The monitoring system consisted of a load cell and static-strain collection box. The steering system consisted of prefabricated bogies. The test piece was securely connected with the beam body by the connection system after it was turned by the steering system. During tensioning, the connection system was used to securely connect it to the tensioning system and real-time data collection was conducted through the external static-strain collection box.

Tensile strength tests of CFRP tendons under bending included the following main steps: (1) Concrete pretreatment, with polishing of the installation area to ensure smoothness; (2) installation into the reaction frame and the bogie on the beam through the planting screw, with a planting depth at 12 cm (≥6 d, d the planting diameter), and completion of connection and installation of all devices; (3) tension, with first pre-tensioning and checking whether the force was uniform through the two sets of load cells arranged at the tension and fixed ends. Then, formal loading was performed until complete failure at a loading speed of 150 MPa/min and the ultimate load obtained.

**Figure 7:**
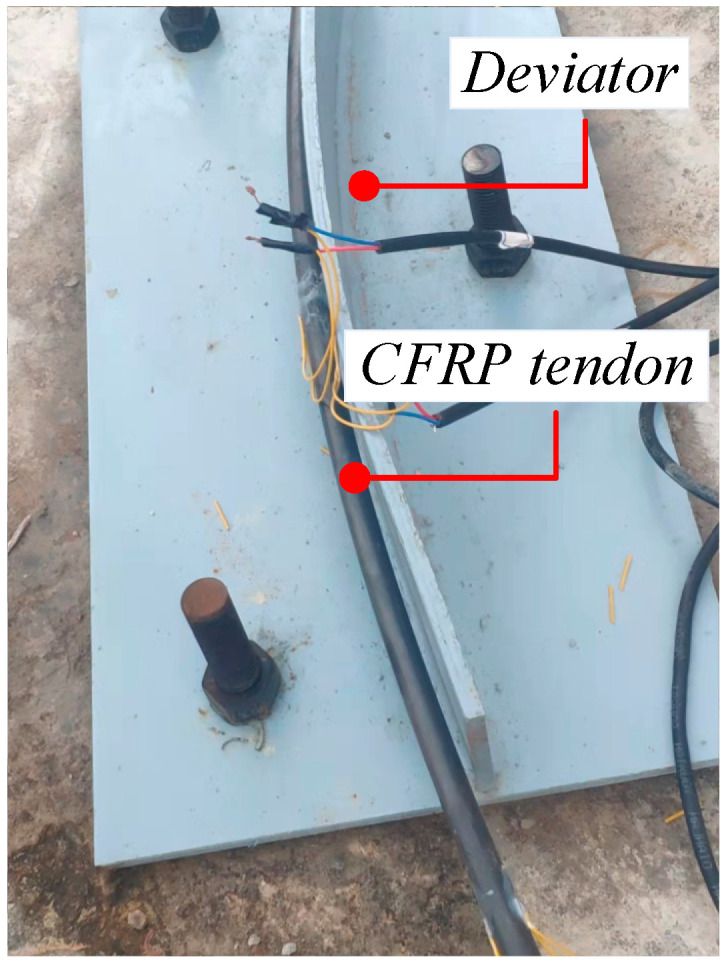
Test part without buffer material.

**Figure 8:**
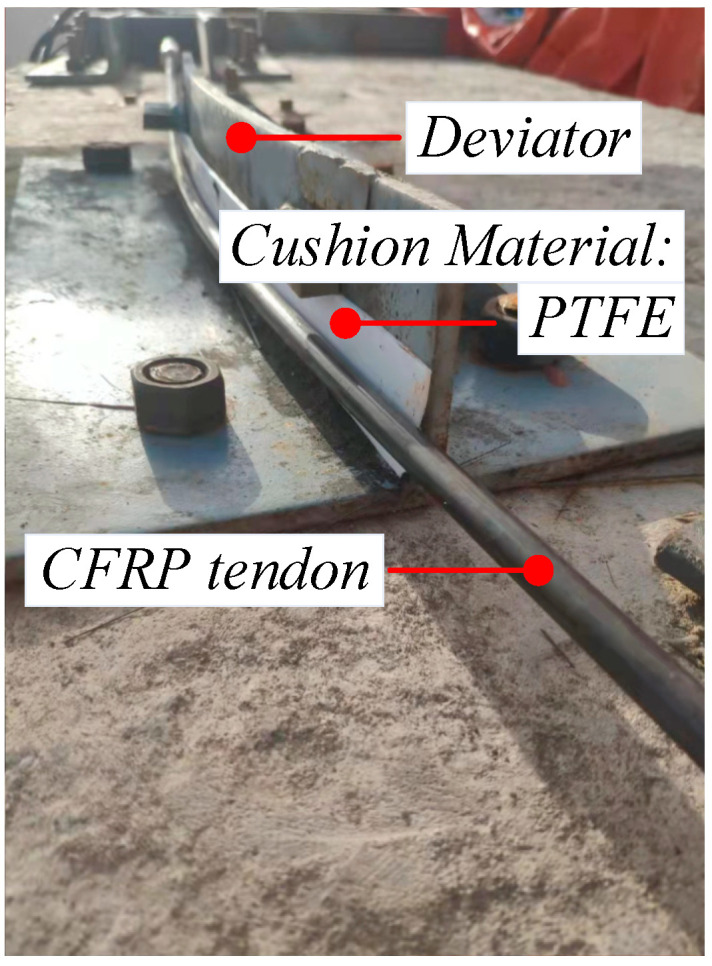
Test part with buffer material.

The first set of tests used CFRP tendons with a diameter of 10 mm as test pieces and the test parameters included the bending angle *θ* and interfacial buffer material. Three different turning angles of 20, 30, and 40° were selected and control conditions without buffer material and with a PTFE plate as buffer material were set (Figure 7 and Figure 8, respectively). The first set of tests involved a total of six sets of working conditions (Table 3).

The second set of tests used three CFRP cables composed of single tendons of 10 mm diameter. Four bending radii of 1.5, 2, 2.5, and 3 m were selected (Figure 6). A total of eight sets of working conditions were set in the second group (Table 4).

#### 3.2.2. Test Results

**Figure 9:**
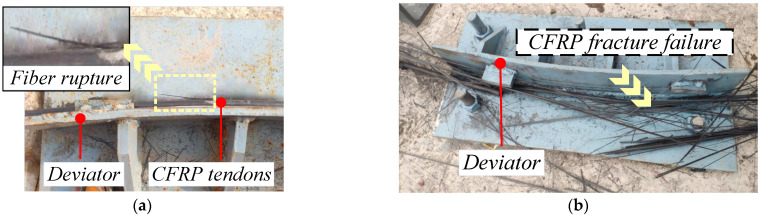
(**a**) Initial failure test, (**b**) Ultimate failure test. Failure in CFRP tendon tests.

According to the theoretical analysis of Section 2.2, the bending stress caused premature tensile damage to the outermost fibers of the CFRP tendon, at which time the section did not fail completely and was in the initial damage state. As the load increased, the fibers continued to break in tension until the section failed completely and reached the ultimate damage state. In the bending test, all specimens failed at the contact tangent position between the CFRP bars and the bogie, exhibiting a tensile damage mode of the fibers. Two stages were observed, including the initial and ultimate failure stages. After initial failure, fibers on the outer side of the tendons ruptured and were out of service, with the stress redistributed in the cross-section (Figure 9a). After further loading, the test pieces underwent continuous fiber fracture until ultimate failure and direct rupture. At this time, the internal force approached 0.00 kN and test pieces experienced uniform explosive failure (Figure 9b).

**Figure 10:**
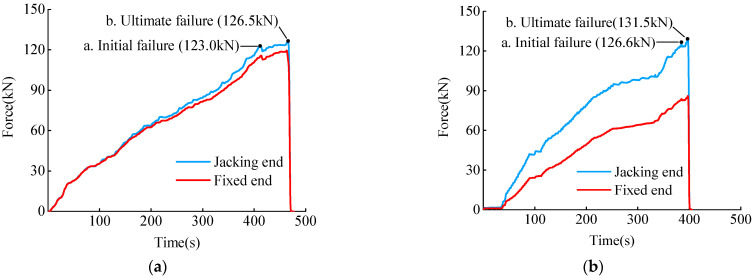
(**a**) Load-time curve for working condition B-20, (**b**) Load-time curve for working condition BF-20. Load-time curve of CFEP tendon tests.

The load-time curve for cases B-20 and BF-20 under single bending showed that two peaks existed on the curve, points a and b (Figure 10), representing the axial force at the tensile end, which corresponded to the initial and the ultimate failure states (Figure 9a,b, respectively). In addition, there was a difference between the axial force curves of the tensioned and fixed ends, and the BF-20 working condition demonstrated a larger gap than the B-20 working condition. This was because BF-20 had no buffer material and larger friction loss than B-20. However, the UBC results of the two sets of working conditions were similar and the same result was also obtained on the basis of comparisons among the working conditions of B-30 and BF30 and of B-40 and BF40. This verified that the bending angle parameter in Equation (7) of the calculation model was not the parameter that influenced the results. Thus, the bending angle and buffer material in the experimental research conditions were not deemed to be factors that affected the ultimate bending capacity.

**Figure 11:**
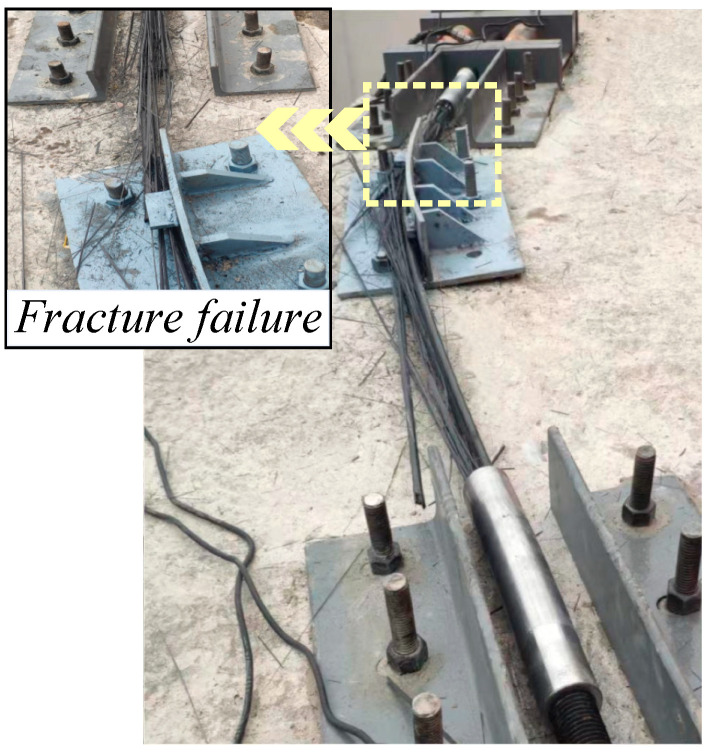
Failure in CFRP cable tests.

Similar to CFRP tendon tests, the failures of CFRP cables all occurred at the tangent position where the cable and bogie contacted. In the bending test, after the initial failure of the CFRP cable, with increased load, the stress of the section was redistributed and the remaining CFRP tendons were unable to bear the original load, at which point the fibers ruptured continuously; finally, the CFRP cable underwent uniform explosive failure at the rupture site (Figure 11).

**Figure 12:**
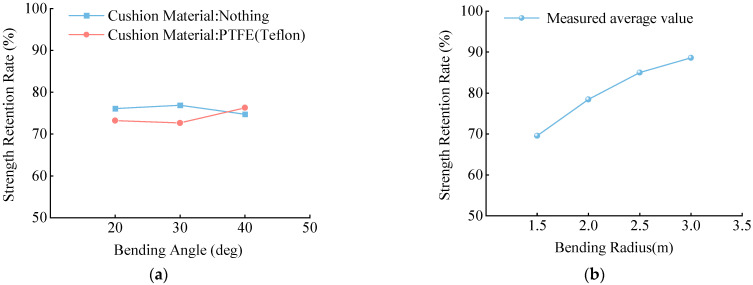
(**a**) Test results of CFRP tendons, (**b**) Test results of CFRP cable. Comparison of test results under different working conditions.

The test results of the CFRP tendons showed that the variation in all results was within 5%, demonstrating high consistency and further verifying that the ultimate bending bearing capacity was not correlated with the bending angle or buffer material (Figure 12a).

The test results of the CFRP cable also showed that, with an increased bending radius, the curves exhibited a clear upward trend, indicating that the bending radius had a significant impact on the strength retention rate (Figure 12b).

#### 3.2.3. Test Verification

The above test results were compared and verified using Equation (7). The ratio of the measured to theoretical values of the UBC under each working condition showed that the measured values of each group were relatively close and consistent with the theoretical analytical results (Table 5). According to the data under all working conditions, the mean ratio *µ* of the experimental to theoretical values was 0.970 and the coefficient of variation *δ* was 0.051 (Table 5). The measured value error under the BF-R1.5-B working condition was great, which might have been caused by an installation accuracy error. Therefore, after removing this working condition, the mean ratio *µ* of the test to theoretical values was 0.981 and the coefficient of variation *δ* dropped to 0.030. The proposed theoretical method was thus concluded to be accurate.

Comparisons between the measured and theoretical values of the strength retention rate under each working condition showed that the bending radius had a significant impact on tensile strength (Table 6). The strength loss was great and more likely to be lower than the theoretical residual strength as a result of an installation error when the bending radius was small. Compared with working conditions with bending radii of 1.5 or 2 m, the strength of a test piece increased by 15.4% and 6.6%, respectively. Moreover, the difference was only 3.6% compared with the working condition with a 3 m bending radius. It was found that, when the bending radius was 2.5 m, the residual strength significantly increased and reached ~85%.

The above tests showed that the bending strength was not affected by the bending angle, the interface buffer material, or the number of CFRP tendons within the cable. When the diameter of the tendons was constant, the bending strength increased with an increased bending radius. The results of the theoretical calculation model of the tensile strength of the cable in the bending state were properly consistent with the experimental results. This basically conformed to the relationship changes of the cable bearing capacity under different working conditions, which also verified the correctness of the theoretical calculation model.

## 4. Discussion and Prediction

### 4.1. Discussion

From a historical point of view, high-strength steel has good mechanical properties, and the use of traditional steel strand and steel cable has led to tremendous developments in bridge engineering; however, its high post-maintenance cost and unstable long-term performance have been major problems for the industry. In comparison, CFRP has a superior performance, its tensile strength is 8–10 times that of steel, and its density is only 1/5 that of steel. Moreover, its corrosion rate is only 1/1000 that of steel, or even lower [1,2]. Therefore, CFRP cables have been considered for bridge structures since 1982 [5]. The first application of CFRP cables in highway bridges was the Stork bridge in Sweden [41]. They have been gradually applied since then, such as in the Neigles pedestrian bridge in Switzerland [42], the Laroin pedestrian bridge in France, and the Penobscot bridge in the United States [43].

With the expansion of applications, problems related to material bending become inevitable. However, scholars have not studied the bending tensile properties of steel strands or cables in depth, because the bending behavior has little effect on their performance. For CFRP ropes, Jerrett et al. [24] directly used bogies of steel strands at the beginning and tested whether the performance was much reduced. They realized the necessity of bending research on CFRP ropes and proceeded to develop bogies suitable for CFRP materials.

In this paper, bending tensile tests were conducted for CFRP ropes at bending angles of 20–40° and turning radii of 1.5–3 m to verify the correctness of the theoretical findings in Section 2. Moreover, we used the calculation model obtained in the previous paper to predict and evaluate the residual strength under actual conditions, so as to provide an intuitive assessment and reference for engineering applications of prestressed CFRP ropes.

### 4.2. Prediction

The research results of previous scholars are often only applicable to their specific materials [35,36,37,38] and cannot be well applied to engineering applications. Furthermore, due to the heterogeneity of material properties, material suppliers can only guarantee the approximate range of material parameters [44]. Therefore, we needed theoretical calculations to predict the error envelope range and thus the ultimate performance. According to the theoretical calculation model (Equation (7)) established in Section 2.2, the influencing factors of the residual tensile strength were the radius of the CFRP tendon *r*, the elastic modulus *E*, the tensile strength *f*, the bending radius *R* of the bogie, and the numerical range of parameters of common reinforcement CFRP tendons [11,12,44,45], with *r* at 3–7 mm, *f* at 2100–2500 MPa, and *E* at 120–160 GPa.

The lower limits of the applicable ranges of Equations (4) and (8) were calculated from the above parameter ranges as *R/r* ≥ 153 and *R/r* ≥ 120, respectively. The value of *R ≥* 200 *r* was conservatively taken, which thus applied to *R ≥* 2 m.

**Figure 13:**
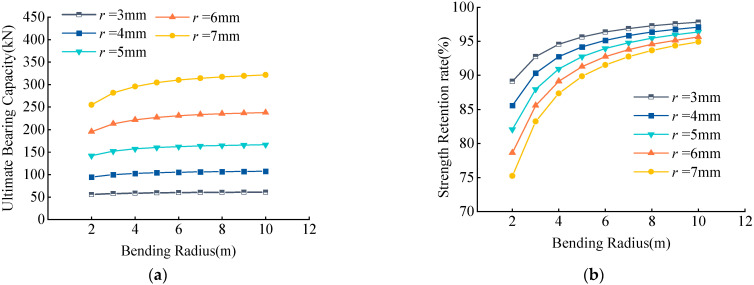
(**a**) Effects of *r* on UBC, (**b**) Effects of *r* on RSR. Effects of radius of carbon fiber tendons *r* on UBC and RSR.

Examination of the variations in the UBC and RSR of CFRP tendons with different radii with bending radius showed that they increased with an increased bending radius (Figure 13). When the turning radius was constant, the UBC increased with increased tendon radius *r*, residual strength decreased with increased radius *r*, and the magnitude of decrease was large.

**Figure 14:**
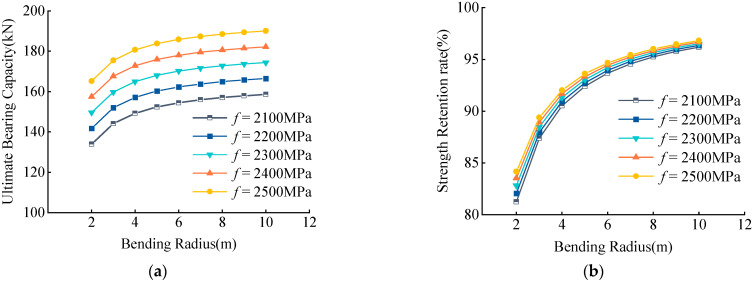
(**a**) Effects of *f* on UBC, (**b**) Effects of *f* on RSR. Effects of tensile strength *f* on UBC and RSR.

Examination of the variation of the UBC and RSR of CFRP tendons with different strengths with bending radius showed that they increased with an increased bending radius (Figure 14). When the bending radius was constant, both the UBC and RSR increased with increased tendon strength f, the residual strength increased by <4%, and the magnitude of increase was small.

**Figure 15:**
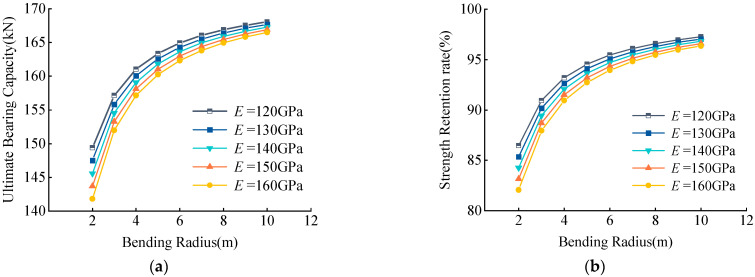
(**a**) Effects of *E* on UBC, (**b**) Effects of *E* on RSR. Effects of elastic modulus *E* on UBC and RSR.

Examination of the variation of the UBC and RSR of CFRP tendons with different elastic modulus with the bending radius showed that they increased with an increased bending radius (Figure 15). When the bending radius was constant, both the UBC and the RSR decreased with an increased elastic modulus, residual strength decreased by less than 6%, and the magnitude of decrease was small.

Within the performance parameters of the common reinforcement CFRP tendons above, *r* > *f* > *E* were ranked by the extent of effects on the UBC (Figure 13, Figure 14 and Figure 15). *E* had minor effects and was ranked as *r* > *E* > *f* by the effects on residual strength, in which the effects of *f* and *E* were minor. Within the range of 2100–2500 MPa in strength and 120–160 Gpa in elastic modulus, when *R* ≥ 3 m and *r* ≤ 7 mm, the residual strength reached over 80%; when *R* ≥ 6 m and *r* ≤ 7 mm, the residual strength reached over 90%; and when *R* ≥ 10 m and *r* ≤ 7 mm, the residual strength reached almost 95%.

**Figure 16:**
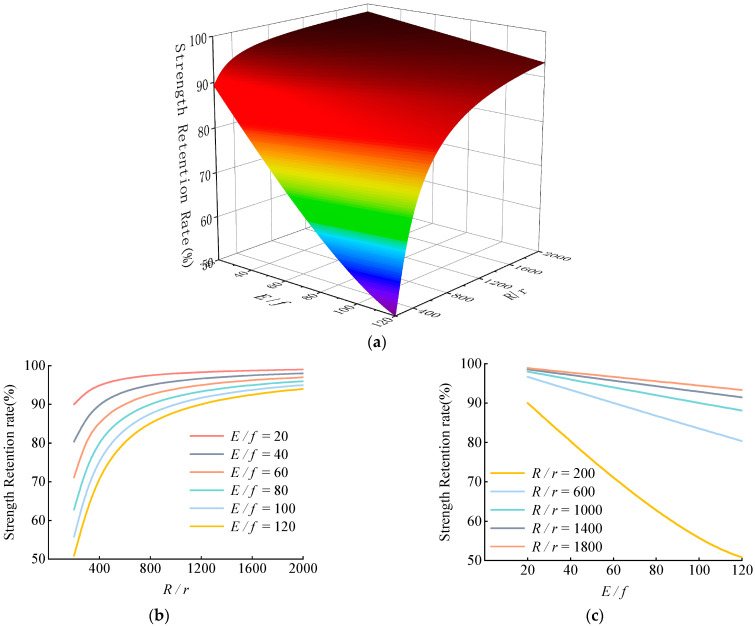
(**a**) Prediction model of residual strength under bending, (**b**) Effects of *R/r* on residual strength, (**c**) Effects of *E/f* on residual strength. Prediction model diagram.

However, a further analysis indicated that the above four parameters were not independent of each other in terms of results. Based on the analysis in Section 2.2, the residual strength of bending tensile was only affected by the quantities of *R/r* and *E/f*. Thus, by referring to Equation (7), a prediction model was obtained (Figure 16).

Within the performance parameters of the common reinforcement CFRP tendons examined above, RSR increased nonlinearly with increased *R/r* (Figure 16). When 200 ≤ *R/r* ≤ 600, this variation was significant; when *R/r* ≥ 600, the increasing trend was not significant; and with the RSR of over 80%, and when *R/r* ≥ 1200, the RSR reached more than 90%. The RSR decreased almost linearly with increased *E/f*. Under the most unfavorable condition, *E/f* = 120, *R/r* = 200, the RSR was only ~50%. It was thus clear that both *R/r* and *E/f* had a great impact on the residual strength and comprehensive consideration should be given to avoid any unfavorable conditions.

In the example of the test material in this study, *E/f* = 72.7, when *R/r* ≥ 800, the RSR reached 90% and, when *R/r* ≥ 1600, the RSR reached 95%. At present, most CFRP tendons with a 5 mm radius are used in actual bending and shear resistance reinforcement projects and the bending radius is generally >5 m, i.e., *R/r* ≥ 1000. Hence, CFRP cables are highly reliable in engineering reinforcement applications due to their low loss in residual strength and high tensioning efficiency.

## 5. Conclusions

To evaluate the tensile strength of CFRP cables under bending, this study conducted theoretical analyses and experimental tests, which revealed the tensile performance and failure mechanism of CFRP cables under bending, and established a residual tensile strength evaluation model based on the internal force failure criterion. The major conclusions were as follows:

(1) Based on theoretical research in Section 2, the tensile failure mode of CFRP cable involved tensile failure of the fiber. Based on the failure criterion of the maximum axial force, the study established an evaluation and calculation model for the tensile strength of CFRP cables under bending and verified the model through test results. This model was applied to a case where the bending radius *R* was ≥2 in practical engineering and could be used to analyze the behavioral characteristics of the initial and ultimate failure of CFRP cables in applications.

(2) The experimental results indicated that the residual tensile strength of CFRP cables was associated with the radius *r*, tensile strength *f*, elastic modulus *E*, and bending radius *R* of CFRP tendons. It was not correlated with the interfacial buffer material or bending angle. The *r* and *R* were found to have great effects on the residual strength.

(3) The discussion and prediction showed that the RSR was only affected by the combination of *R/r* and *E/f*, it decreased with increased *E/f*, and it increased with increased *R/r*. When *E/f* involved conventional material parameters, *R/r* ≥ 600, the RSR reached more than 80%, and when *R/r* ≥ 1200, the RSR reached >90%. As a result, *R/r* = 600 may be utilized as a basis in the design of a safe critical radius.

## Figures and Tables

**Table 1:** Critical number *n* under the two failure modes.

Bending Angles/m	*n*	*a*	Failure Mode
0.7 (Critical Radius)	≤5550	≤87	1
	≥5550	≥87	2
1	≤16,500	≤149	1
	≥16,500	≥149	2
1.5	≤92,800	≤352	1
	≥92,800	≥352	2
2	≤272,400	≤603	1
	≥272,400	≥603	2

**Table 2:** Static load test data of the CFRP cable.

Test Conditions	Specification/mm	UBC *F* /kN	Average Value of UBC F /kN
S-1	3 × 10	494.6	492.1
S-2	3 × 10	513.9	
S-3	3 × 10	467.7	

**Table 3:** Bending test conditions of CFRP tendons.

Test Conditions	Bending Radii *R* /m	Bending Angles *θ* /°	Cushion Material	Specification /mm
BF-20	1.5	20	/	1 × 10
B-20			PTFE	1 × 10
BF-30		30	/	1 × 10
B-30			PTFE	1 × 10
BF-40		40	/	1 × 10
B-40			PTFE	1 × 10

**Table 4:** Bending test conditions of CFRP cables.

Test Conditions	Bending Radii *R* /m	Bending Angles *θ* /°	Cushion Material	Specification /mm
BF-R1.5-A	1.5	20	/	3 × 10
BF-R1.5-B				3 × 10
BF-R2-A	2			3 × 10
BF-R2-B				3 × 10
BF-R2.5-A	2.5			3 × 10
BF-R2.5-B				3 × 10
BF-R3-A	3			3 × 10
BF-R3-B				3 × 10

Note: Bending angle and interface parameters in working conditions in Table 4 determined and supplemented according to the test results in Table 3.

**Table 5:** Test and theoretical values of bending bearing capacity.

Test Conditions	Test Value /kN	Theoretical Value /kN	Test Value and Theoretical Value Ratio /%
BF-20	131.5	131.3	1.002
B-20	126.5		0.963
BF-30	132.8		1.011
B-30	125.5		0.956
BF-40	129.1		0.983
BF-40	131.8		1.004
BF-R1.5-A	360.6	393.9	0.915
BF-R1.5-B	326.2		0.828
BF-R2-A	401.4	424.5	0.946
BF-R2-B	411.6		0.970
BF-R2.5-A	445.2	443.1	1.005
BF-R2.5-B	435.9		0.984
BF-R3-A	457.7	455.7	1.004
BF-R3-B	460.7		1.011

**Table 6:** Residual tensile strength under bending.

Test Conditions	$\eta_{t}$ (%)	${\bar{\eta }}_{t}$ (%)	η (%)
BF-20	76.10	74.65	75.98
B-20	73.21		
BF-30	76.85	74.74	
B-30	72.63		
BF-40	74.71	75.49	
BF-40	76.27		
BF-R1.5-A	69.57	69.57	75.99
BF-R1.5-B	62.93		
BF-R2-A	77.44	78.42	81.89
BF-R2-B	79.40		
BF-R2.5-A	85.89	84.99	85.48
BF-R2.5-B	84.09		
BF-R3-A	88.30	88.59	87.91
BF-R3-B	88.88		

Note: *η_t_* and *η* are measured mean values of the retention rate of residual tensile strength and theoretical values of retention rate of tensile residual strength, respectively. Removal condition BF-R1.5-B.

## Data Availability

The data used to support the findings of this study are available from the corresponding author upon request.
